# Circ-PDE1C/miR-766-3p/SGTB axis regulates the IL-1β-induced apoptosis, inflammation and oxidative stress in human chondrocytes

**DOI:** 10.1186/s42358-024-00429-0

**Published:** 2024-12-30

**Authors:** Lixia Gao, Tao He, Qingkui Hu, Yan Ma

**Affiliations:** 1 Department of Rehabilitation Medicine, Wuhan No.1 Hospital, 215 Zhongshan Avenue, Qiaokou District, Wuhan, Hubei 430022 China; 2 College of Sports Medicine, Wuhan Sports University, Wuhan, Hubei 430079 China

**Keywords:** Circ-PDE1C, miR-766-3p, SGTB, Chondrocytes, Osteoarthritis

## Abstract

**Background:**

Osteoarthritis (OA) is a common degenerative joint disease. Circular RNA Phosphodiesterase 1 C (circ-PDE1C, hsa_circ_0134111) has participated in the IL-1β-induced chondrocyte damages. The objective of our study was to explore the molecular mechanism of circ-PDE1C.

**Methods:**

Circ-PDE1C, microRNA-766-3p (miR-766-3p) or Small Glutamine Rich Tetratricopeptide Repeat Co-Chaperone Beta (SGTB) expression was determined using reverse transcription-quantitative polymerase chain reaction (RT-qPCR). Cell counting kit-8 (CCK-8) assay and flow cytometry were used to analyze proliferation and apoptosis, respectively. Western blotting assay was performed for protein detection. The inflammatory cytokines were measured by Enzyme-linked immunosorbent assay (ELISA). Oxidative stress was assessed by commercial kits. Target analysis was conducted by dual-luciferase reporter assay and RNA immunoprecipitation (RIP) assay.

**Results:**

Circ-PDE1C was abnormally overexpressed in OA tissues and IL-1β-exposed chondrocytes. Downregulation of circ-PDE1C alleviated the IL-1β-induced cell apoptosis, inflammation, extracellular matrix degradation and oxidative stress. Circ-PDE1C could interact with miR-766-3p to serve as miRNA sponge. The function of si-circ-PDE1C was attributed to the inhibition of miR-766-3p. Additionally, miR-766-3p directly targeted the 3’UTR of SGTB. The miR-766-3p upregulation impeded the IL-1β-triggered cell damages through reducing the level of SGTB. Moreover, SGTB expression was regulated by circ-PDE1C via binding to miR-766-3p in IL-1β-induced chondrocytes.

**Conclusion:**

Altogether, circ-PDE1C enhanced the IL-1β-induced dysfunction in chondrocytes via upregulating SGTB by targeting miR-766-3p.

**Supplementary Information:**

The online version contains supplementary material available at 10.1186/s42358-024-00429-0.

## Highlights

Inhibition of circ-PDE1C partly prevents the IL-1β-induced apoptosis, inflammation and oxidative injury.

IL-1β-induced OA progression is inhibited by circ-PDE1C/miR-766-3p axis and miR-766-3p/SGTB axis.

Circ-PDE1C can elevate the level of SGTB via sponging miR-766-3p.

**Supplementary Information:**

The online version contains supplementary material available at 10.1186/s42358-024-00429-0.

## Introduction

Osteoarthritis (OA) has become a leading cause of disability in many adults [1]. OA is characterized by inflammation and progressive degradation of articular cartilages [2]. Thus, chondrocytes play important roles in the pathological process of OA [3]. The production of pro-inflammatory cytokines, such as IL-1β, can induce apoptotic and oxidative damages in chondrocytes [4]. The diagnosis and treatment remain to be improved in OA [5]. The pathogenic mechanism of IL-1β in OA remains to be explored.

The noncoding circular RNAs (circRNAs) are covalently closed-loop transcripts with absence of 5’ and 3’ polarity [6]. A mass of circRNAs are differentially expressed in OA cartilage tissues and implicated in the OA pathogenesis through microRNA (miRNA) sponge effect to induce gene regulation [7]. CircRNA-9119 relieved IL-1β-aroused cell apoptosis in chondrocytes by inhibiting microRNA-26a and upregulating PTEN [8]. Circ-CSNK1G1 was associated with chondrocyte apoptosis and extracellular matrix (ECM) degradation through the miR‑4428-mediated FUT2 level elevation [9]. CircRSU1 facilitated inflammatory response and oxidative injury via acting on miR-93-5p/MAP3K8 axis [10].

Zhang et al. reported that circRNA Phosphodiesterase 1 C (circ-PDE1C, hsa_circ_0134111) aggravated the symptom of OA through depending on miR-224-5p/CCL2 axis [11]. Also, Wu et al. found that circ-PDE1C mitigated the IL-1β-evoked cell injury via miR-515-5p/SOCS1 axis [12]. IL-1β-caused dysfunction was inhibited by microRNA-766-3p (miR-766-3p) in human chondrocytes [13, 14]. SGTB knockdown was shown to alleviate chondrocyte apoptosis in OA [15, 16]. It is interesting to investigate whether circ-PDE1C can regulate the SGTB level via absorbing miR-766-3p.

This study researched the potential of circ-PDE1C targeting miR-766-3p and miR-766-3p targeting SGTB. The key point was to validate circ-PDE1C/miR-766-3p/SGTB signal axis in the progression of OA cell model, intending to elucidate the specific molecular mechanism of circ-PDE1C.

## Materials and methods

### Tissue acquisition

OA cartilage tissues (*n* = 40) and normal cartilage tissues (*n* = 40) were collected at Wuhan No.1 Hospital. The tissues were identified by histopathological examination through two experienced pathologists. These tissues were saved in liquid nitrogen for RNA isolation. 80 written informed consent files have been provided before this study was performed. Meanwhile, this study was ratified by Ethics Committee of Wuhan No.1 Hospital.

### Cell culture and IL-1β exposure

CHON-001 cell line was bought from BioVector NTCC Inc. (Beijing, China). The basic RPMI-1640 medium (Gibco, Carlsbad, CA, USA) was added with 10% FBS (Gibco) and 1% antibiotics (Gibco) for the preparation of nutrient solution. Then cells were cultured at 37°C under 5% CO_2_. 10 ng/mL IL-1β (Sigma, St. Louis, MO, USA) was incubated to CHON-001 cells for 24 h to induce cell inflammation and injury.

### Cell transfection

SiRNA of circ-PDE1C (si-circ-PDE1C), miR-766-3p mimic, miR-766-3p inhibitor, and si-NC, mimic NC, inhibitor NC were all acquired from RIBOBIO (Guangzhou, China). The pcDNA 3.1 vector (GENESEED, Guangzhou, China) was cloned with the coding sequence of SGTB, then the pcDNA 3.1-SGTB (SGTB) was applied for overexpression of SGTB. CHON-001 cells with 70% confluence were performed with transfection of RNAs or plasmids employing Lipofectamine™ 3000 Kit (Invitrogen, Carlsbad, CA, USA) in line with the guidance.

### RT-qPCR assay

TRI Reagent (Sigma) was utilized for total RNA extraction. Next, 2 µg RNA was transcribed into cDNA through Reverse Transcription Kit (Applied Biosystems, Foster City, CA, USA). PCR detection was conducted by PowerUp™ SYBR™ Green Master Mix (Applied Biosystems). Meanwhile, miRNA level was determined by TaqMan Advanced miRNA cDNA Synthesis Kit and TaqMan™ MicroRNA Assay Kit (Applied Biosystems). Afterwards, relative expression analysis was performed using 2^−∆∆Ct^ method [17]. For level normalization, GAPDH and U6 were employed as the endogenous references. In addition, circ-PDE1C stability was assessed by RT-qPCR after RNA digestion with RNase R (4 U/µg, GENESEED) for 1 h. Also, nuclear or cytoplastic RNA was isolated by PARIS™ Kit (Invitrogen) followed by RT-qPCR detection for localization analysis. The used primers were synthesized by Sangon (Shanghai, China), as shown in Table 1.

**Table 1:** Primer sequences used for RT-qPCR

Name	Primers for PCR (5’-3’)	
circ-PDE1C	Forward	CAGCAACAGAATGGTGACTTCAA
	Reverse	TCTTCAGCCTGGCTTTTGGC
miR-766-3p	Forward	GCCGAGACTCCAGCCCCACAGC
	Reverse	CTCAACTGGTGTCGTGGAG
SGTB	Forward	ACTCAGTGCCTGAAGATGTGG
	Reverse	TGCAACACCCTCCCTAATCAC
PDE1C	Forward	GCCGCTTTTCTCTCCCTTCT
	Reverse	TGGACTCCAGCTCCAAGAGA
GAPDH	Forward	GACAGTCAGCCGCATCTTCT
	Reverse	GCGCCCAATACGACCAAATC
U6	Forward	CTCGCTTCGGCAGCACA
	Reverse	AACGCTTCACGAATTTGCGT

### CCK-8 assay

1 × 10^4^ cells/well were planted into 48-well plates for 18 h. Then cells were exposed to IL-1β and transfected with RNA or vector for 24 h. Each well of plates was pipetted with 10 µL CCK-8 (Sangon), then cells were incubated at 37°C for 3 h. Whereafter, we measured OD value at λ = 450 nm via a microplate reader.

### Flow cytometry

3 × 10^5^ cells were collected for apoptosis examination via Annexin V Apoptosis Kit (Sangon). In brief, cells in 1 × Binding Buffer were stained with 5 µL Annexin V-FITC and 10 µL PI. After lucifugal incubation for 30 min, cell analysis under flow cytometer (BD Biosciences, San Diego, CA, USA) was performed to distinguish apoptotic cells (Annexin V^+^/PI^−^, Annexin V ^+^/PI^+^).

### Western blotting

The proteins were extracted from tissues or cells via RIPA buffer (Sangon). After the concentration was detected by BCA Assay kit (Sangon), expression detection with 50 µg/sample was performed as previously reported [18]. The primary antibodies (1:1000) and secondary antibody (1:5000) were acquired from Abcam (Cambridge, UK). The protein blots were shown by EasyBlot ECL kit (Sangon), and protein levels were analyzed through ImageJ software. The primary antibodies were exhibited as follows: PCNA (ab18197), Cleaved caspase-3 (ab2302), Aggrecan (ab3778), SGTB (ab202419), β-actin (ab8227/ab8224). β-actin served as a housekeeping gene to correct expression.

### ELISA

Inflammatory response was assessed by determining the release of TNF-α and IL-6. The operating procedures followed the guidelines of TNF alpha Human ELISA Kit and IL-6 Human ELISA Kit (Invitrogen).

### Oxidative assays

The assessment of oxidative damage was administrated applying with commercial kits. As per the users’ manuals, ROS and MDA levels were examined ROS Detection Kit and MDA Assay Kit (KeyGen, Nanjing, China). In addition, the activity of superoxide dismutase (SOD) was measured using Total SOD Detection Kit (KeyGen).

### Dual-luciferase reporter assay

Circ-PDE1C/miR-766-3p and miR-766-3p/SGTB site binding was predicted by Circinteractome and Targetscan. The sequences of circ-PDE1C and 3’UTR of SGTB were constructed into pmirGLO (Promega, Madison, WI, USA), respectively. These positive plasmids were considered as WT plasmids (WT-circ-PDE1C, WT-SGTB 3’UTR). Meanwhile, miR-766-3p binding region in circ-PDE1C or SGTB 3’UTR sequence was mutated. Then MUT plasmids (MUT-circ-PDE1C, MUT-SGTB 3’UTR) were used as control groups. The monolayer CHON-001 cells in the 48-well plates were transfected with luciferase plasmid and mimic NC or miR-766-3p mimic. Luciferase activity determination by Dual-luciferase Reporter Detection Kit (Promega) was performed after 48 h.

### RIP assay

Target analysis was further implemented employing Imprint^®^ RNA Immunoprecipitation Kit (Sigma). The magnetic beads were enveloped with anti-AGO2, using anti-IgG as the control group. Subsequently, the beads were co-incubate with 1 × 10^6^ CHON-001 cells at 4°C overnight. The total RNA was purified by TRI Reagent (Sigma), and the molecule levels (circ-PDE1C, miR-766-3p, SGTB) were quantified via RT-qPCR.

### Statistical analysis

SPSS 22.0 (SPSS Inc., Chicago, IL, USA) was exploited for statistical analysis of data (mean ± standard deviation). The assays have been performed for three times with three parallel samples (*n* = 3). Difference was assessed by Student’s *t*-test for two groups, and ANOVA followed by Tukey’s test was used for difference analysis among multiple groups. *P* value less than 0.05 was defined as a significant difference.

## Results

### Circ-PDE1C was highly expressed in OA tissues

**Fig. 1:**
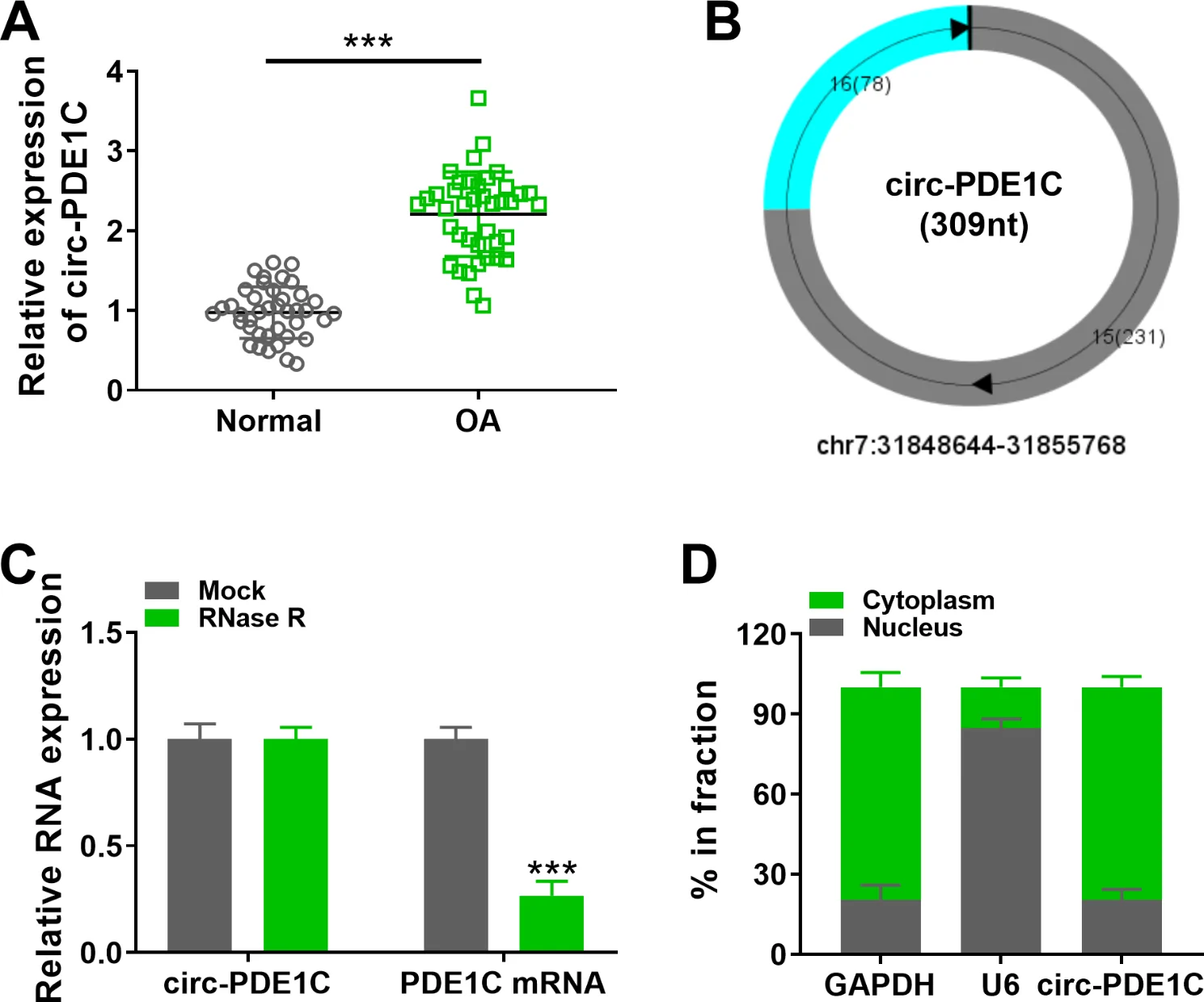
Circ-PDE1C was highly expressed in OA tissues. **A** RT-qPCR was performed for circ-PDE1C expression examination. **B** The genic information of circ-PDE1C. **C** RNase R treatment and RT-qPCR were performed for stability detection of circ-PDE1C and PDE1C in CHON-001 cells. **D** RT-qPCR was performed for localization analysis of circ-PDE1C. ***P < 0.001

The data from RT-qPCR showed that circ-PDE1C expression was much higher in OA tissue samples than that in normal controls (Fig. 1A). Circ-PDE1C (hsa_circ_0134111) is produced by backsplicing of exon 15–16 in PDE1C gene, and its length is 309 nt with the location on the chr7: 31,848,644–31,855,768 (Fig. 1B). PDE1C mRNA level was downregulated by RNase R treatment but no significant difference was found in circ-PDE1C level, which suggested that circ-PDE1C was more stable than linear PDE1C in CHON-001 cells (Fig. 1C). GAPDH and U6 were used as control genes for cytoplasm and nucleus, then circ-PDE1C was identified to be located in the cytoplasm of CHON-001 cells (Fig. 1D). The preliminary detection demonstrated that circ-PDE1C was dysregulated in OA.

### IL-1β-induced cell apoptosis, inflammation and oxidative stress were alleviated with the knockdown of circ-PDE1C

**Fig. 2:**
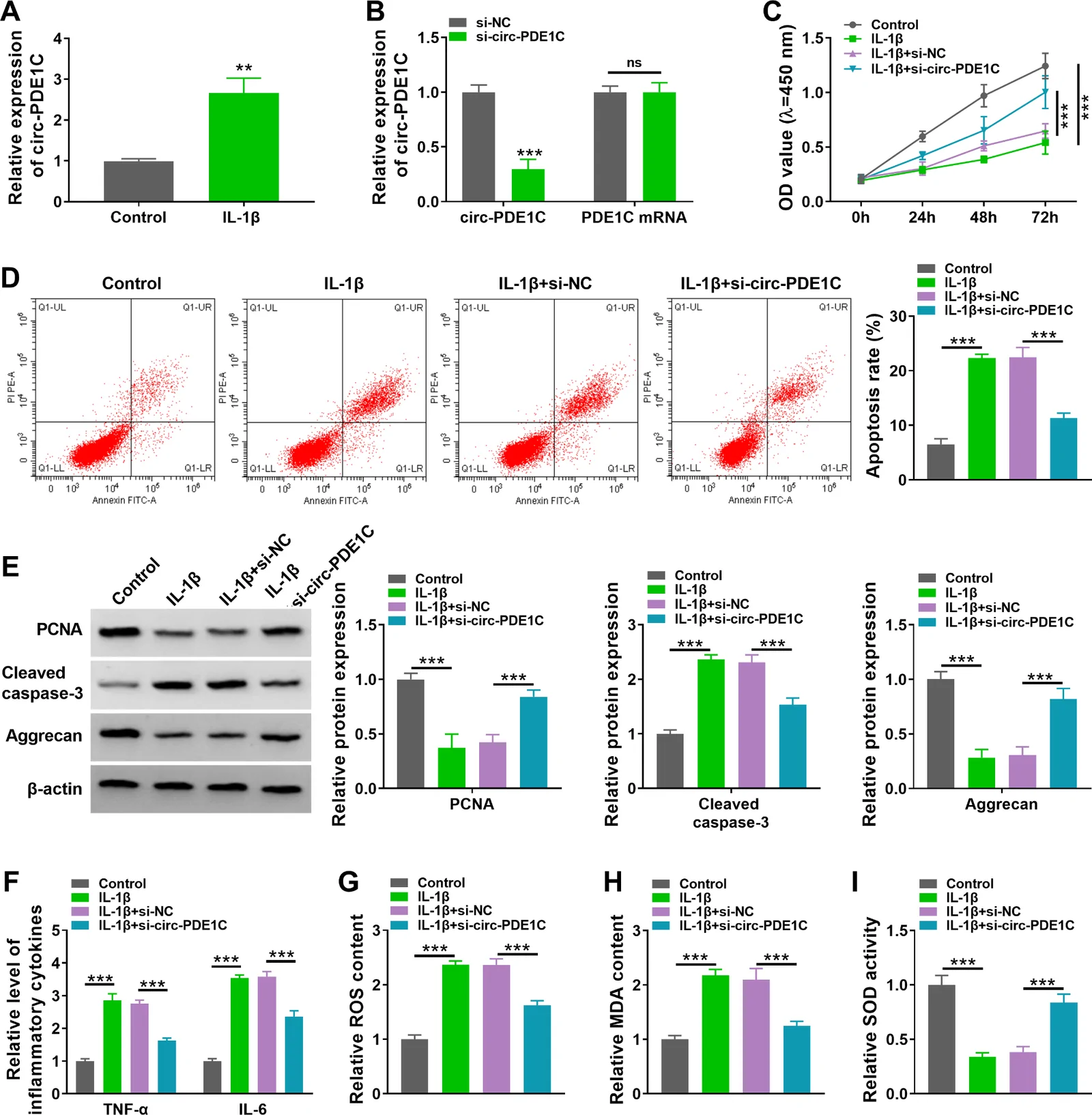
IL-1β-induced cell apoptosis, inflammation and oxidative stress were alleviated with the knockdown of circ-PDE1C. **A** The circ-PDE1C expression was detected by RT-qPCR after CHON-001 cells were treated with IL-1β. **B** The circ-PDE1C and PDE1C mRNA levels were measured in si-NC or si-circ-PDE1C transfected CHON-001 cells. **C** Cell proliferation was assessed by CCK-8 assay in control, IL-1β, IL-1β + si-NC, and IL-1β + si-circ-PDE1C groups. **D** The apoptosis rate was determined by flow cytometry. **E** PCNA, Cleaved caspase-3 and Aggrecan protein levels were examined by western blotting. **F** TNF-α and IL-6 levels were assayed by ELISA. **G**–**I** Oxidative stress was evaluated by the detection of ROS (**G**), MDA (**H**) and SOD (**I**). **P < 0.01, ***P < 0.001

IL-1β treatment also resulted in the circ-PDE1C upregulation in CHON-001 cells relative to control group (Fig. 2A). Then, the function of circ-PDE1C was investigated after circ-PDE1C expression was knocked down in CHON-001 cells. The knockdown effect of si-circ-PDE1C was conspicuous in circ-PDE1C level but not PDE1C mRNA level (Fig. 2B). IL-1β-induced the inhibition of cell proliferation (Fig. 2C) and the increase of apoptosis rate (Fig. 2D) were eliminated by transfection of si-circ-PDE1C. Western blot results indicated that silence of circ-PDE1C promoted the protein levels of PCNA (proliferation marker) and Aggrecan (ECM marker) but reduced the protein expression of Cleaved caspase-3 (apoptotic marker) in IL-1β-treated CHON-001 cells (Fig. 2E). By performing ELISA detection, we found that si-circ-PDE1C inhibited the release of TNF-α and IL-6 caused by IL-1β (Fig. 2F). ROS and MDA levels were elevated (Fig. 2G-H) while SOD activity was decreased (Fig. 2I) in IL-1β-treated CHON-001 cells, then circ-PDE1C downregulation abolished the oxidative stress. Overall, circ-PDE1C expression reduction partly mitigated the IL-1β-induced cell injury.

### Circ-PDE1C was confirmed as a mir-766-3p inhibitor

**Fig. 3:**
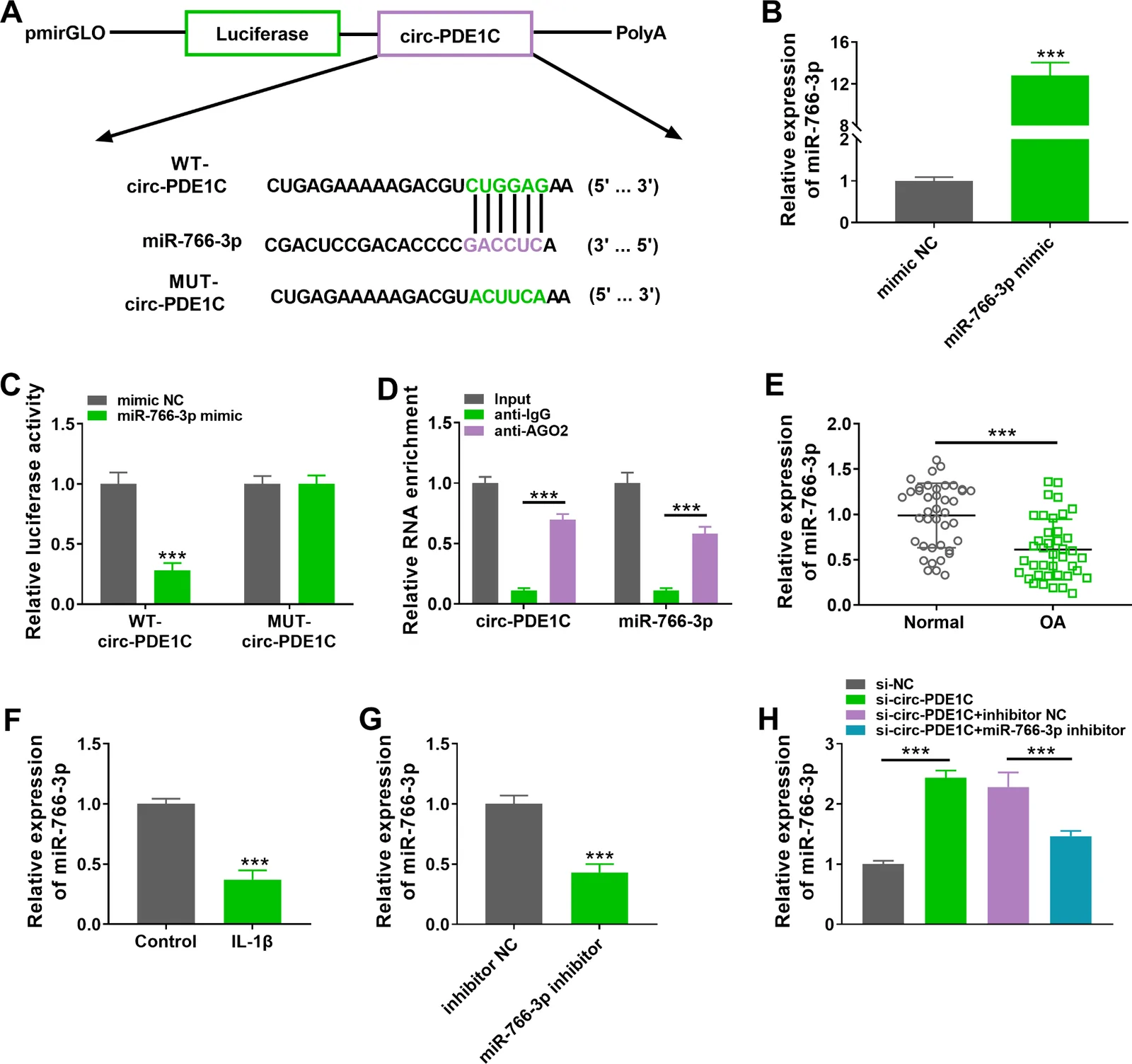
Circ-PDE1C was confirmed as a miR-766-3p inhibitor. **A** The binding sites between circ-PDE1C and miR-766-3p were predicted using circinteractome. **B** The overexpression efficiency of miR-766-3p mimic was determined using RT-qPCR. **C**, **D** The combination between circ-PDE1C and miR-766-3p was validated using dual-luciferase reporter assay (**C**) and RIP assay (**D**). **E**, **F** The level of miR-766-3p was examined using RT-qPCR in OA tissues (**E**) and IL-1β-treated CHON-001 cells (**F**). **G** The inhibitory effect of miR-766-3p inhibitor on miR-766-3p level was analyzed using RT-qPCR. **H** The expression of miR-766-3p was quantified using RT-qPCR after transfection of si-NC, si-circ-PDE1C, si-circ-PDE1C + inhibitor NC or si-circ-PDE1C + miR-766-3p inhibitor. ***P < 0.001

Circinteractome precited that circ-PDE1C contained a binding site of miR-766-3p (Fig. 3A). WT and MUT luciferase plasmids were constructed for dual-luciferase reporter assay. RT-qPCR has revealed that miR-766-3p level was obviously overexpressed by miR-766-3p mimic, contrasted to mimic NC group (Fig. 3B). The results of luciferase detection manifested that there was an inhibitory effect of miR-766-3p overexpression on luciferase activity of WT-circ-PDE1C plasmid, instead of MUT-circ-PDE1C plasmid (Fig. 3C). Circ-PDE1C and miR-766-3p were enriched in anti-AGO2 group relative to anti-IgG group, which also confirmed the interaction between circ-PDE1C and miR-766-3p (Fig. 3D). The level of miR-766-3p was markedly downregulated in OA samples (Fig. 3E) and IL-1β-treated CHON-001 cells (Fig. 3F), compared with normal samples and control cells. The transfection efficiency of miR-766-3p inhibitor was evident in CHON-001 cells (Fig. 3G), and inhibition of miR-766-3p reversed the si-circ-PDE1C-mediated expression upregulation of miR-766-3p (Fig. 3H). Therefore, circ-PDE1C could induce the direct expression inhibition by binding to miR-766-3p.

### The protective function of si-circ-PDE1C was suppressed by mir-766-3p inhibitor in IL-1β-treated CHON-001 cells

**Fig. 4:**
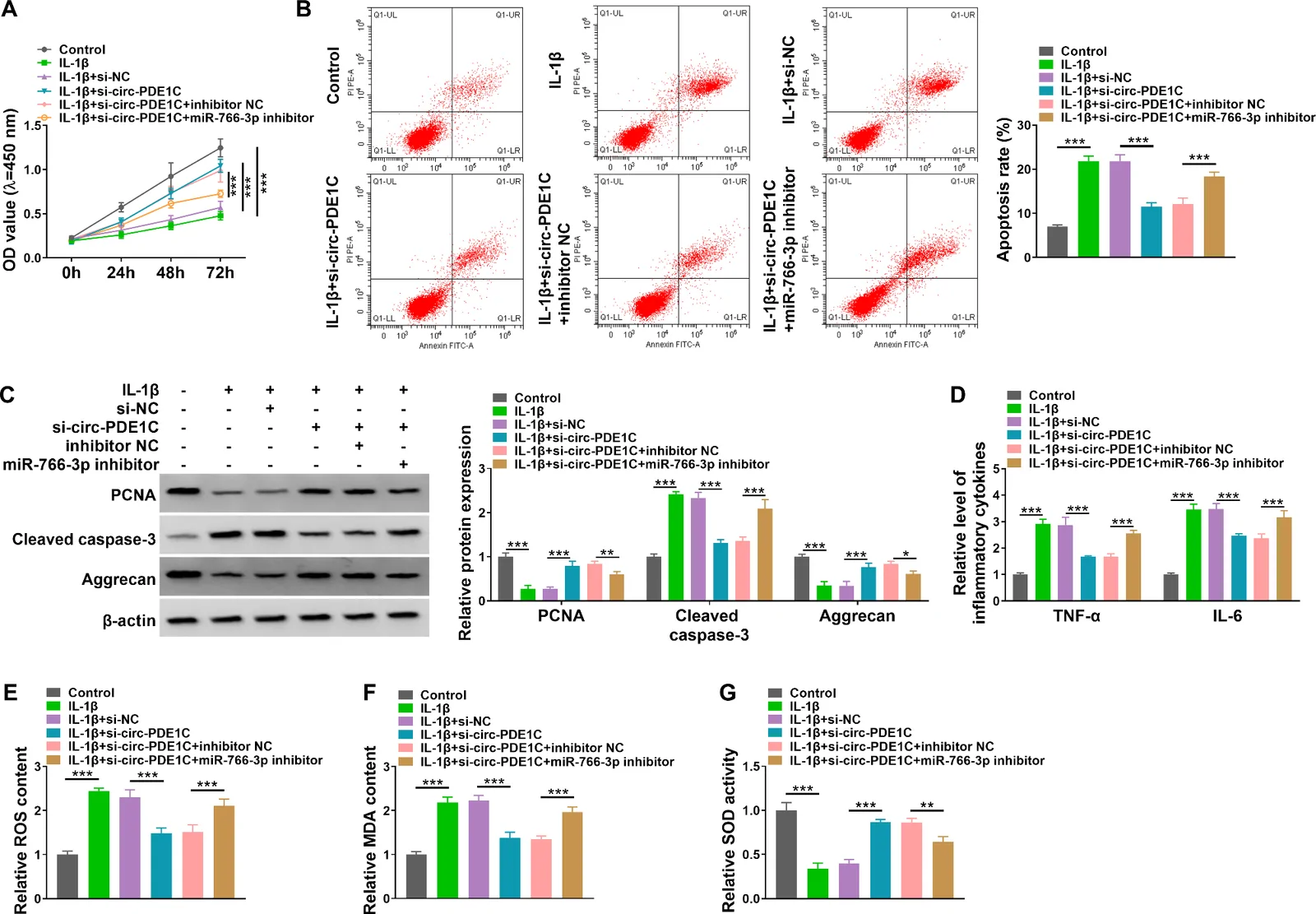
The protective function of si-circ-PDE1C was suppressed by miR-766-3p inhibitor in IL-1β-treated CHON-001 cells. CHON-001 cells were treated with control, IL-1β, IL-1β + si-NC, IL-1β + si-circ-PDE1C, IL-1β + si-circ-PDE1C + inhibitor NC or IL-1β + si-circ-PDE1C + miR-766-3p inhibitor. **A** CCK-8 assay was used to measure cell proliferation. **B** Flow cytometry was used to assess cell apoptosis. **C** Western blotting assay was used to examine the protein expression of PCNA, Cleaved caspase-3 and Aggrecan. **D** ELISA was used to determine the levels of TNF-α and IL-6. **E**–**G** ROS level (**E**), MDA level (**F**) and SOD activity (**G**) were detected by commercial kits. *P < 0.05, **P < 0.01, ***P < 0.001

The further experiments were performed to explore the association of miR-766-3p with circ-PDE1C function in CHON-001 cells. The reversal effects of si-circ-PDE1C on cell proliferation (Fig. 4A) and apoptosis (Fig. 4B) in IL-1β-treated CHON-001 cells were counteracted following the downregulation of miR-766-3p. Meanwhile, transfection of miR-766-3p inhibitor abrogated the expression changes of PCNA, Cleaved caspase-3 and Aggrecan induced by si-circ-PDE1C in IL-1β-stimulated cells (Fig. 4C). Also, immunofluorescence result showed that higher fluorescence intensity of Aggrecan was aroused by si-circ-PDE1C in IL-1β-induced chondrocytes, then miR-766-3p downregulation mitigated this regulation (Supplementary Fig. 1). As the results of miR-766-3p inhibition, the si-circ-PDE1C-mediated suppression of inflammatory response (Fig. 4D) and oxidative stress (Fig. 4E-G) was offset under the treatment of IL-1β. These results suggested that the role of circ-PDE1C was associated with the negative regulation of miR-766-3p in IL-1β-induced cell injury.

### SGTB was a target gene of miR-766-3p

**Fig. 5:**
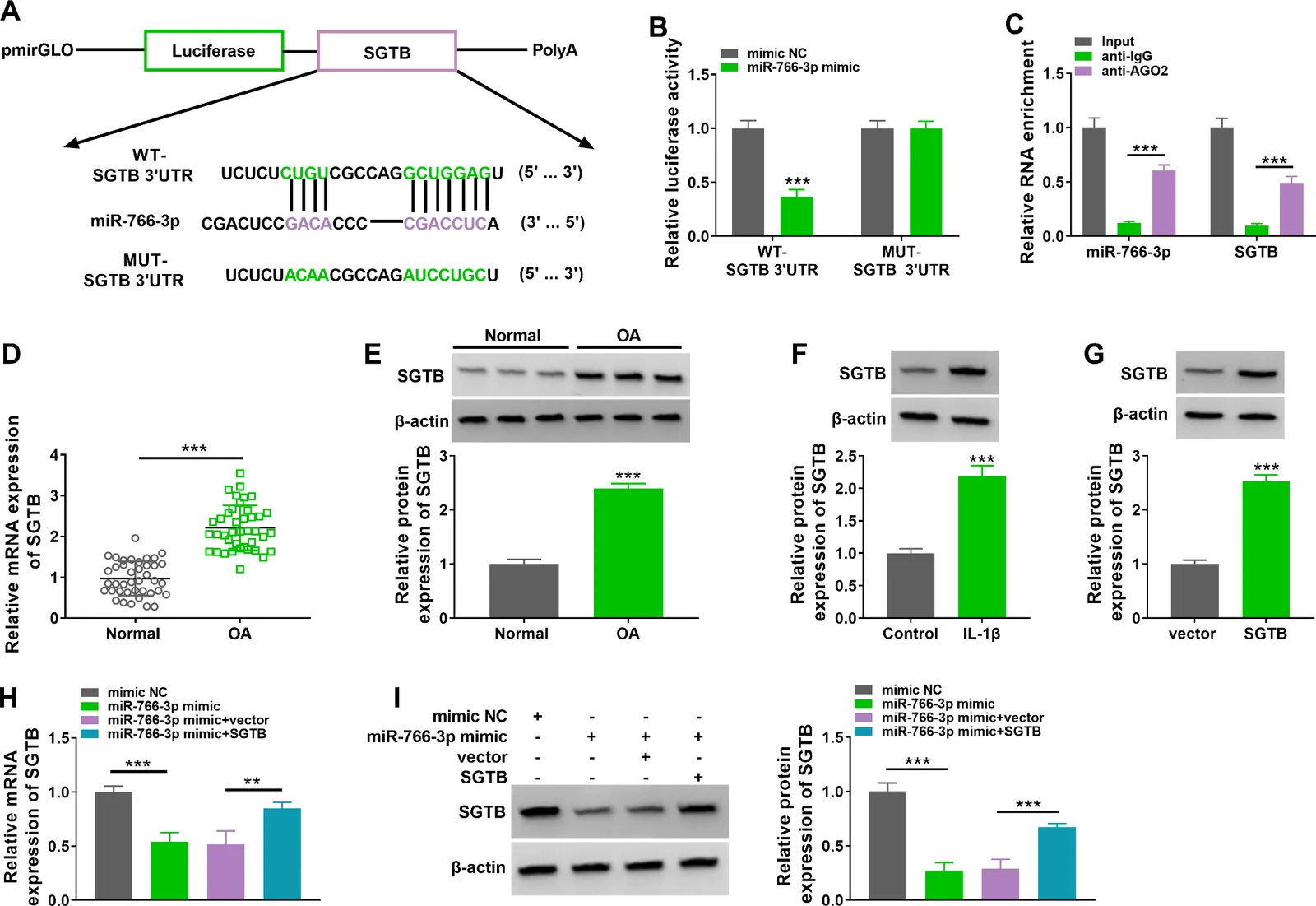
SGTB was a target gene of miR-766-3p. **A** Targetscan exhibited the binding sites between SGTB 3’UTR and miR-766-3p. **B**, **C** Dual-luciferase reporter assay (**B**) and RIP assay (**C**) were conducted to affirm the binding between miR-766-3p and SGTB 3’UTR. **D**, **E** RT-qPCR and western blotting were conducted to detect the mRNA and protein levels of SGTB in OA tissues. **F** SGTB protein expression analysis was conducted by western blotting in IL-1β-exposed CHON-001 cells. **G** Transfection efficacy of SGTB was evaluated through western blot. **H**, **I** SGTB mRNA and protein determination was carried out by RT-qPCR and western blotting in mimic NC, miR-766-3p mimic, miR-766-3p mimic + vector and miR-766-3p mimic + SGTB groups. **P < 0.01, ***P < 0.001

The binding sites of miR-766-3p were observed in the SGTB 3’UTR sequence by Targetscan (Fig. 5A). Subsequently, miR-766-3p was affirmed to combine with SGTB through the dual-luciferase reporter assay (Fig. 5B) and RIP assay (Fig. 5C). RT-qPCR and western blotting assays demonstrated that SGTB expression was increased in cartilage tissues from OA patients compared with the normal tissues (Fig. 5D-E). SGTB protein level was also upregulated by IL-1β treatment in CHON-001 cells (Fig. 5F). Relative to vector transfection, SGTB transfection led to the significant upregulation of SGTB protein expression (Fig. 5G). Overexpression of miR-766-3p reduced the mRNA and protein levels of SGTB, which was ameliorated by the introduction of SGTB (Fig. 5H-I). The above findings showed that miR-766-3p directly targeted SGTB.

### IL-1β evoked cell damages in CHON-011 cells via the regulation of miR-766-3p/SGTB axis

**Fig. 6:**
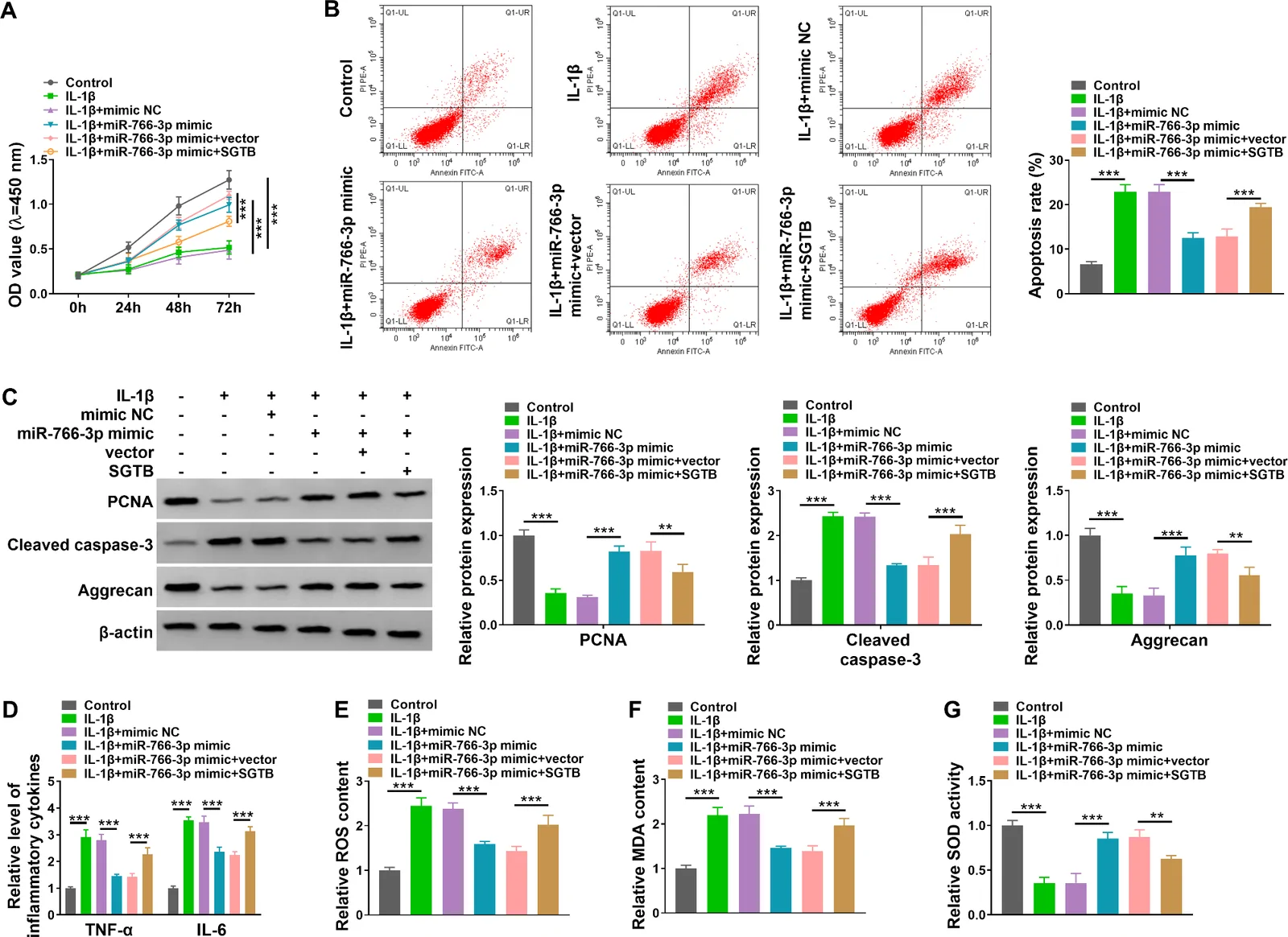
IL-1β evoked cell damages in CHON-011 cells via the regulation of miR-766-3p/SGTB axis. The control, IL-1β, IL-1β + mimic NC, IL-1β + miR-766-3p mimic, IL-1β + miR-766-3p mimic + vector or IL-1β + miR-766-3p mimic + SGTB. **A** The proliferation detection was conducted via CCK-8 assay. **B** Flow cytometry was performed for apoptosis analysis. **C** The protein examination of PCNA, Cleaved caspase-3 and Aggrecan was performed via western blotting. **D** ELISA was applied for TNF-α and IL-6 determination. **E**–**G** The assessment of oxidative stress was performed via the measurement of ROS level (**E**), MDA level (**F**) and SOD activity (**G**). **P < 0.01, ***P < 0.001

Whether the miR-766-3p/SGTB axis was involved in the IL-1β-induced cell injury was researched. The miR-766-3p mimic increased cell proliferation (Fig. 6A) and repressed cell apoptosis (Fig. 6B), while these effects were returned by transfection of SGTB in IL-1β-treated CHON-001 cells. SGTB overexpression also relieved the protein upregulation of PCNA and Aggrecan but the protein downregulation of Cleaved caspase-3 caused by miR-766-3p mimic after IL-1β treatment (Fig. 6C). In addition, the IL-1β-induced inflammatory cytokine release (Fig. 6D) and oxidative stress (Fig. 6E-G) were attenuated by miR-766-3p mimic via downregulating the level of SGTB. Taken together, miR-766-3p could lighten the IL-1β-induced cell dysfunction by targeting SGTB.

### Circ-PDE1C regulated the SGTB level by interacting with mir-766-3p in IL-1β-induced CHON-011 cells

**Fig. 7:**
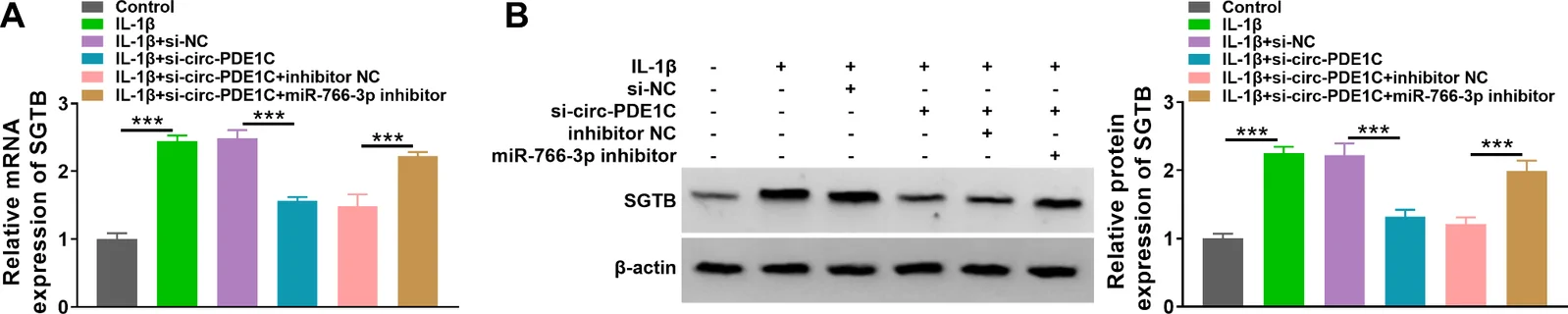
Circ-PDE1C regulated the SGTB level by interacting with miR-766-3p in IL-1β-induced CHON-011 cells. **A**, **B** The mRNA and protein levels of SGTB were examined through RT-qPCR and western blotting in control, IL-1β, IL-1β + si-NC, IL-1β + si-circ-PDE1C, IL-1β + si-circ-PDE1C + inhibitor NC or IL-1β + si-circ-PDE1C + miR-766-3p inhibitor group. ***P < 0.001

RT-qPCR and western blotting were performed to analyze the expression effect of circ-PDE1C on SGTB. The data revealed that si-circ-PDE1C abated the promoting impacts of IL-1β on SGTB mRNA and protein levels, whereas this regulation was abolished by miR-766-3p inhibitor in CHON-011 cells (Fig. 7A-B). Thus, circ-PDE1C positively regulated the SGTB expression via targeting miR-766-3p in IL-1β-treated CHON-001 cells.

## Discussion

The present evidence demonstrated that circ-PDE1C promoted chondrocyte injury caused by IL-1β in OA. Additionally, circ-PDE1C function was partly related to the level changes of miR-766-3p and SGTB. These findings unraveled that circ-PDE1C/miR-766-3p/SGTB axis could affect the pathological process of OA.

IL-1β signaling has central effects on the function of chondrocytes in OA [19]. IL-1β can evoke cell inflammation and reduce the synthesis of cartilage ECM [20]. Our results exhibited that IL-1β restrained proliferation but aggravated cell apoptosis. Aggrecan protein downregulation demonstrated that IL-1β exposure resulted in the degradation of ECM. The detection of inflammatory cytokines and oxidative indicators manifested that IL-1β enhanced inflammation and oxidative stress in chondrocytes. IL-1β has resulted in severe damages in human chondrocytes.

The novel circRNAs are known to act as vital regulators in OA. For instance, circSERPINE2 attenuated cell apoptosis and improved ECM anabolism in OA progression [21]. Ni et al. discovered that circPSM3 reduced cell proliferation and differentiation in OA chondrocytes [22]. CircVCAN induced apoptosis promotion and proliferation inhibition in OA [23]. Herein, the expression analysis revealed that circ-PDE1C was overexpressed in OA samples. Silence of circ-PDE1C has facilitated proliferation ability but reduced apoptosis occurrence, ECM degradation, inflammatory reaction and oxidative injury under the IL-1β treatment. All those effects of IL-1β have been abolished by circ-PDE1C downregulation. Hence, circ-PDE1C functioned as a promoter in OA progression.

The functional regulation of circRNA is usually related to the sponge effect on miRNA in human diseases, including OA [7]. CircRNA.33,186 contributed to the OA progression via the sponge function of miR-127-5p [24]. CircRNA-CDR1as promoted ECM metabolism and inflammatory response through inhibiting miR-641 [25]. In this research, circ-PDE1C induced miR-766-3p level reduction via miRNA sponging function. Furthermore, miR-766-3p downregulation recovered the protective function of si-circ-PDE1C. These data implied that circ-PDE1C was implicated in OA pathogenesis through sequestering miR-766-3p.

MiRNAs affect the disease progression by regulating the gene expression. Also, the anti-pathogenic influences of miR-766-3p were owed to the AIFM1 and DNMT3A level repression in OA [13, 14]. Our results affirmed the targe relation of miR-766-3p with SGTB, and SGTB overexpression abated the miR-766-3p-induced protection from IL-1β treatment in human chondrocytes. In addition, circ-PDE1C knockdown led to the level downregulation of SGTB via affecting the miR-766-3p level. Silencing Circ-SPG11 has relieved the IL-1β-induced chondrocyte injury in OA through depending on the miR-1277/TRAF6 axis [26]. Circ_0020093 attenuated the IL-1β-aroused ECM degradation and chondrocyte apoptosis via upregulating SPRY1 through serving as a miR-23b sponge [27]. Also, the circ-PDE1C function was achieved partly via modulating the miR-766-3p/SGTB network.

## Conclusion

**Fig. 8:**
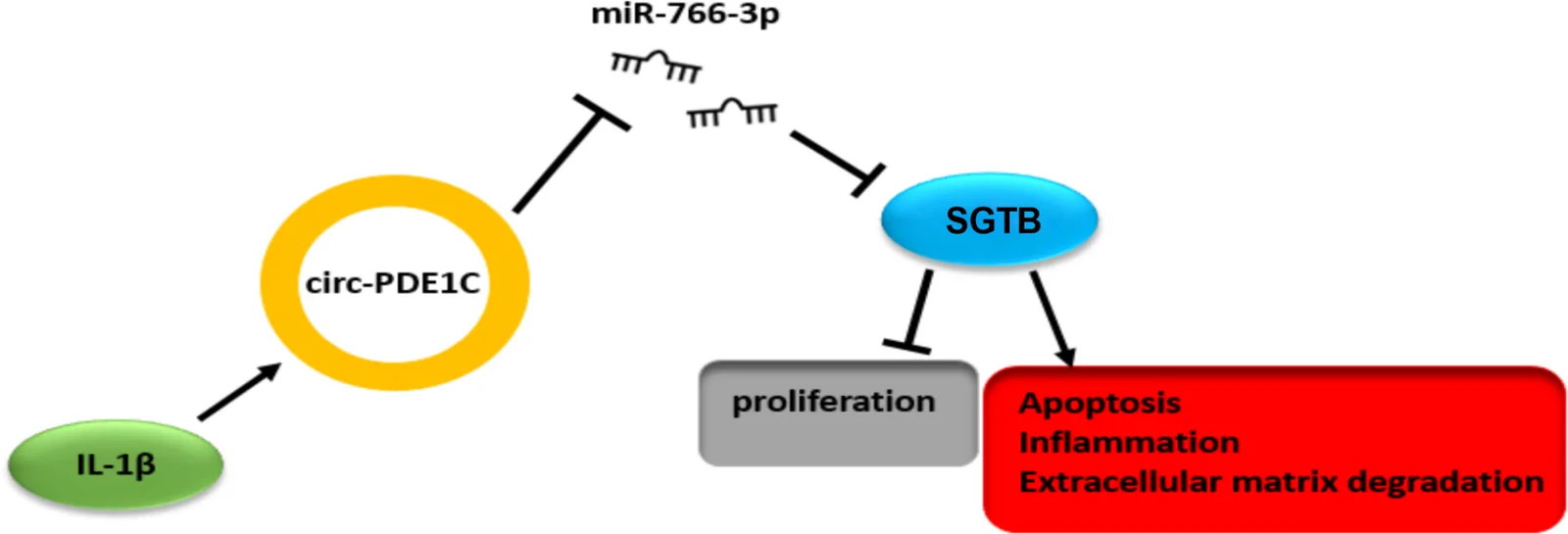
Graphical abstract of the current study

In summary, circ-PDE1C targeted miR-766-3p to regulate the SGTB level to affect the IL-1β-triggered chondrocyte injury (Fig. 8). This study confirmed the protective action of circ-PDE1C downregulation in IL-1β-stimulated OA model, and provided a novel regulatory mechanism for circ-PDE1C. These results might contribute to the circRNA research in OA progression.

## Electronic supplementary material

Below is the link to the electronic supplementary material.

Supplementary Material 1: **Fig. 1** Immunofluorescence analysis for Aggrecan in groups of Fig. 4

Supplementary Material 2

## Data Availability

The analyzed data sets generated during the present study are available from the corresponding author on reasonable request.
