# Supplementary material for: Circ-PDE1C/miR-766-3p/SGTB axis regulates the IL-1β-induced apoptosis, inflammation and oxidative stress in human chondrocytes
Source: Adv Rheumatol. 2024 Dec 30;64:92. doi: 10.1186/s42358-024-00429-0 (PMC13488740; doi:10.1186/s42358-024-00429-0)

Fig.2E

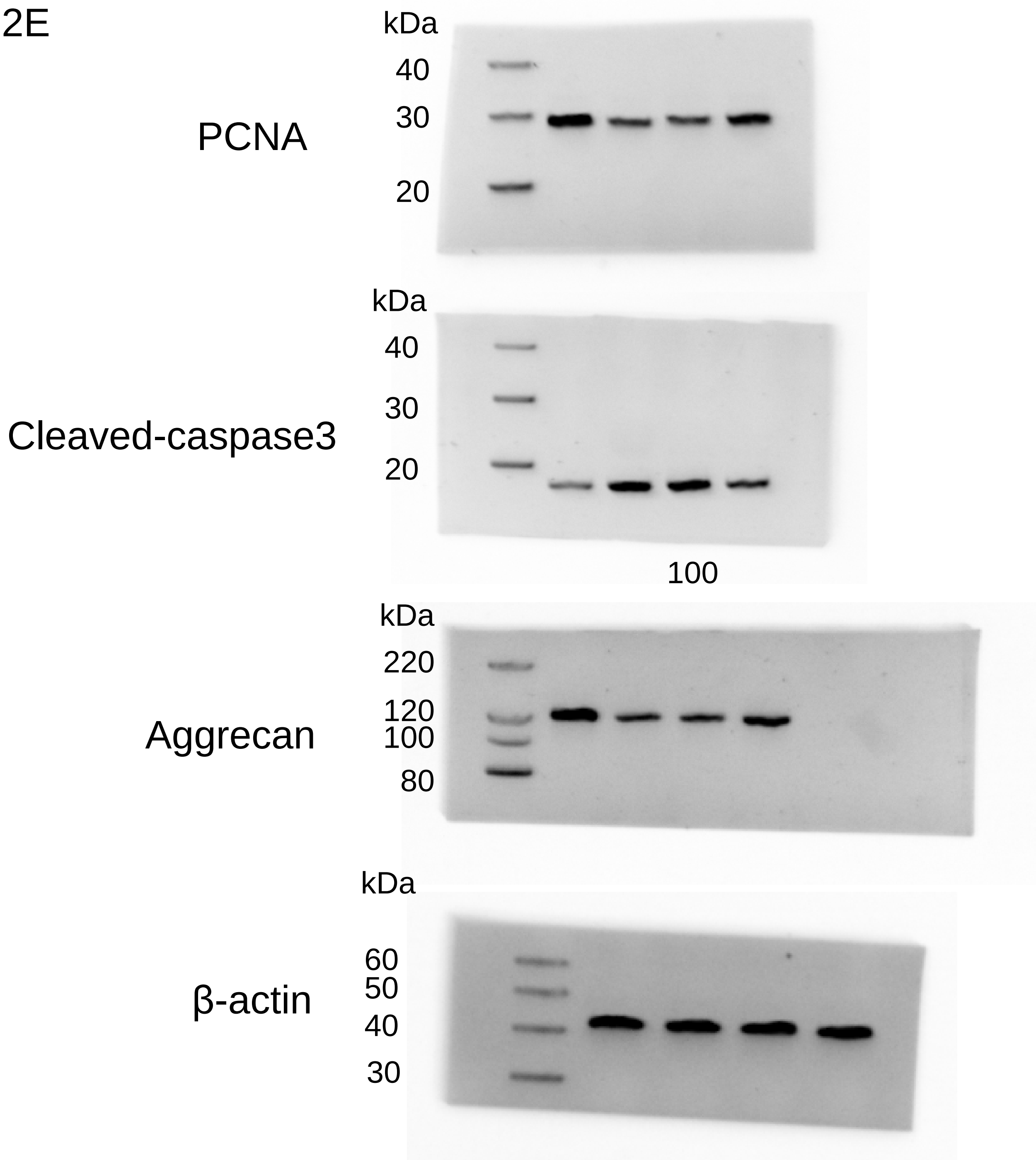
kDa
40
30
20
PCNA
kDa
40
30
20
Cleaved-caspase3
100
kDa
220
120
100
80
Aggrecan
kDa
60
50
40
30
β-actin

Fig.4C

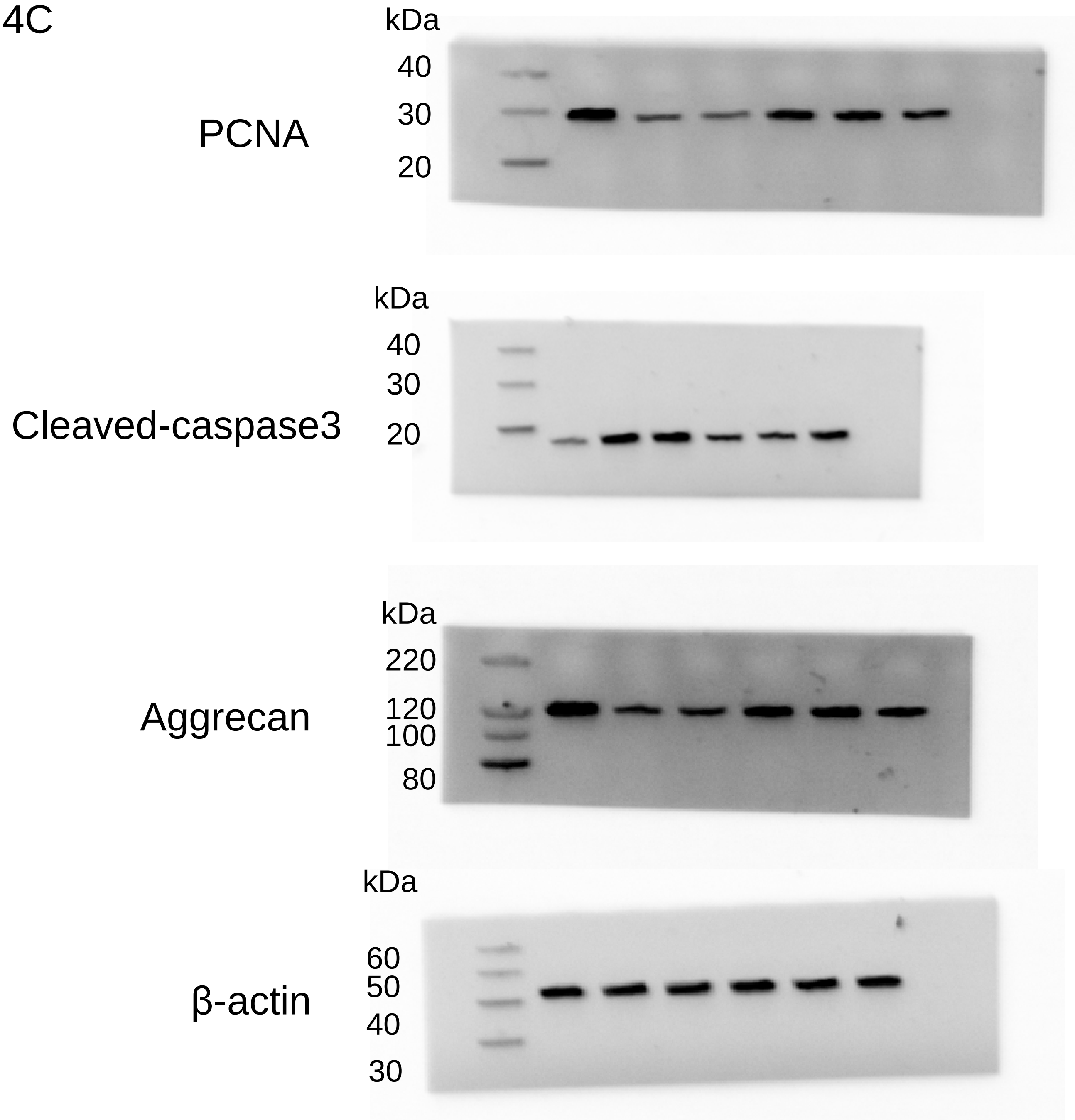

PCNA
kDa
40
30
20
Cleaved-caspase3
kDa
40
30
20
Aggrecan
kDa
220
120
100
80
β-actin
kDa
60
50
40
30

Fig.5E

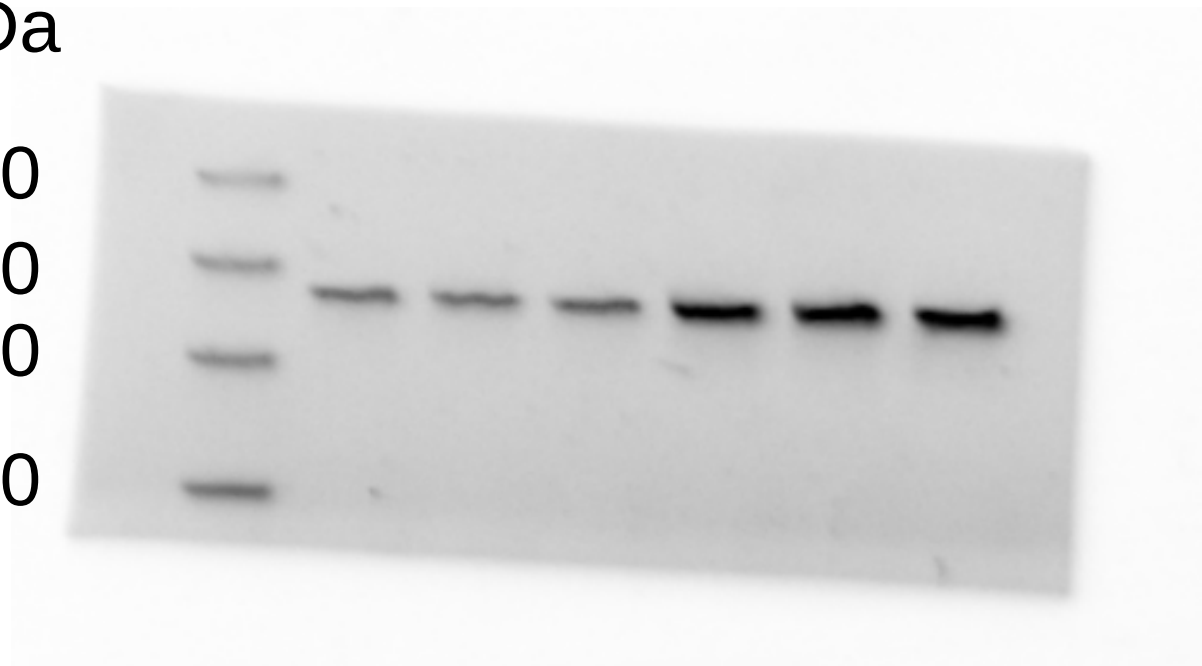

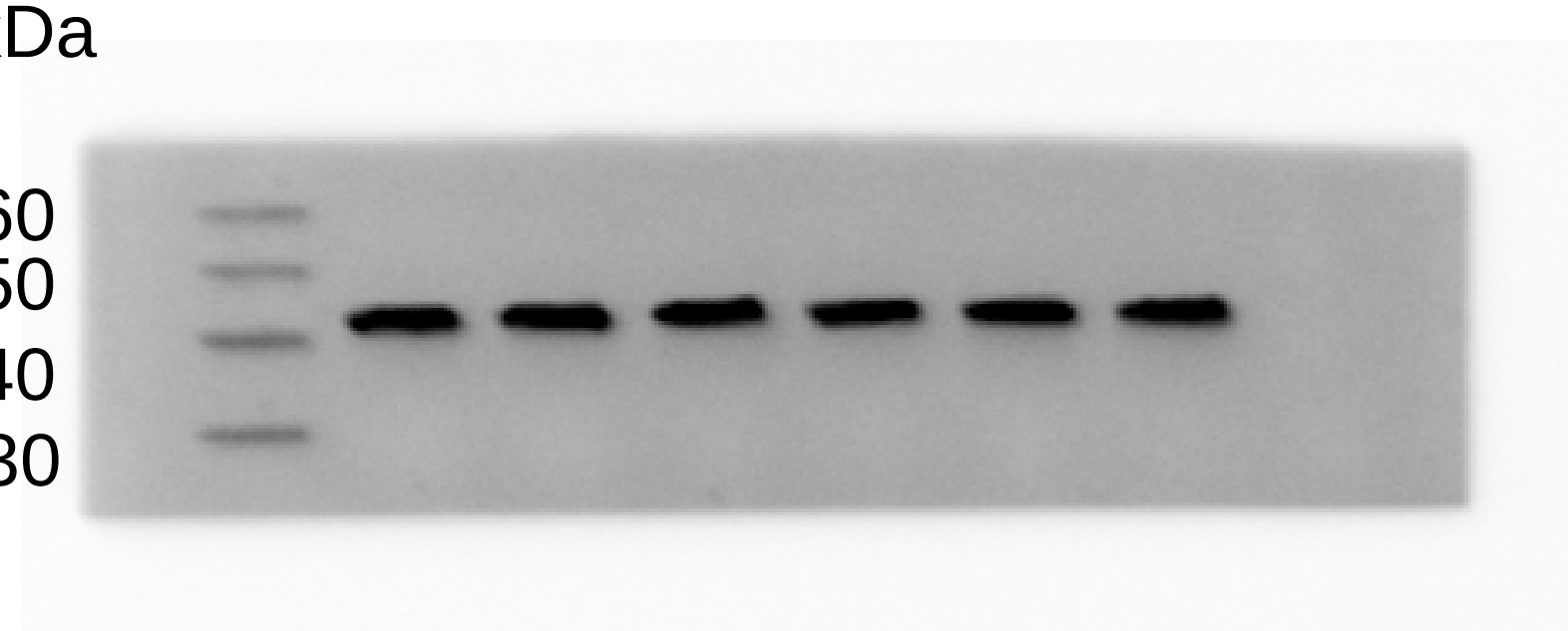

Fig.5F

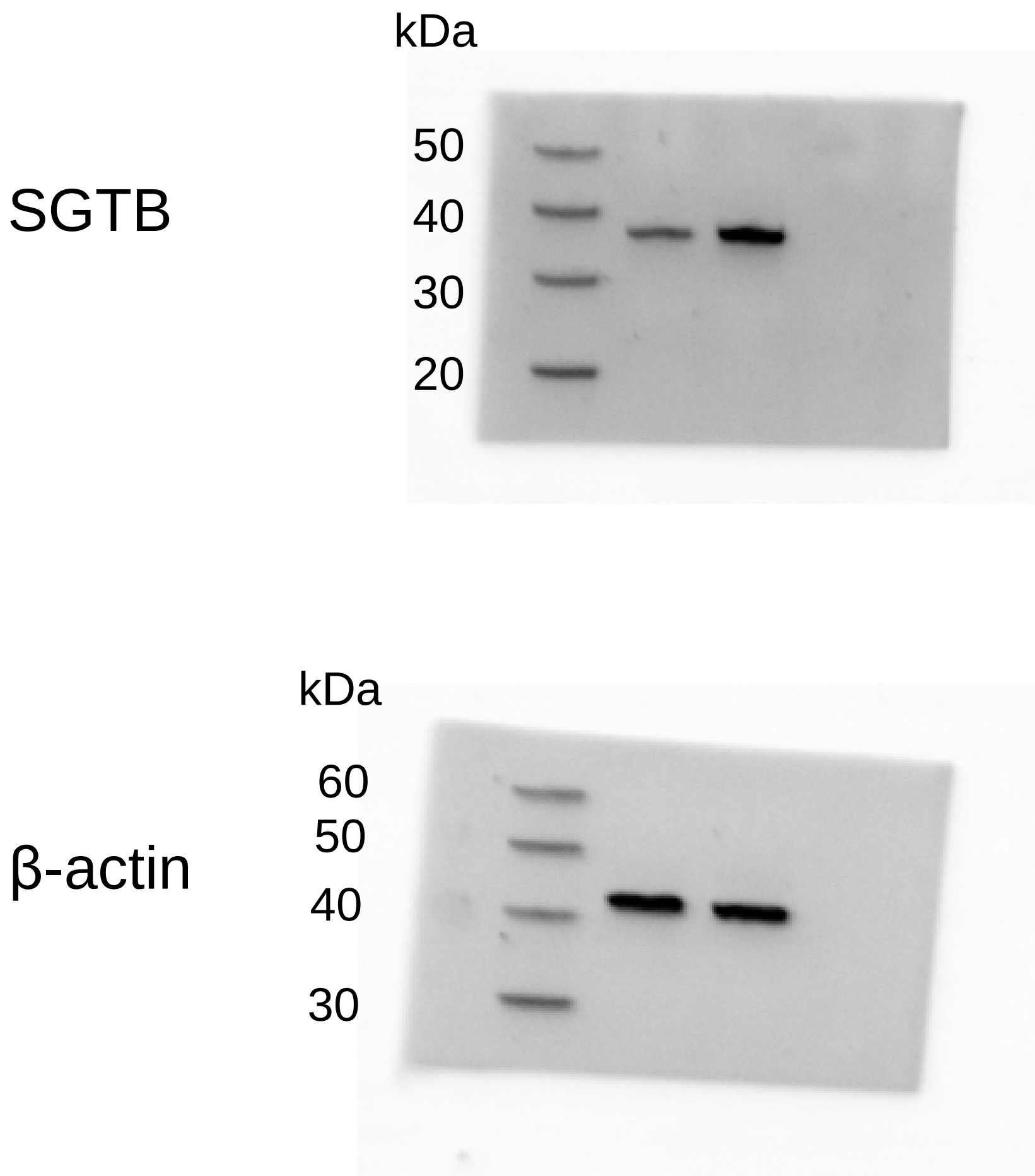

Fig.5G

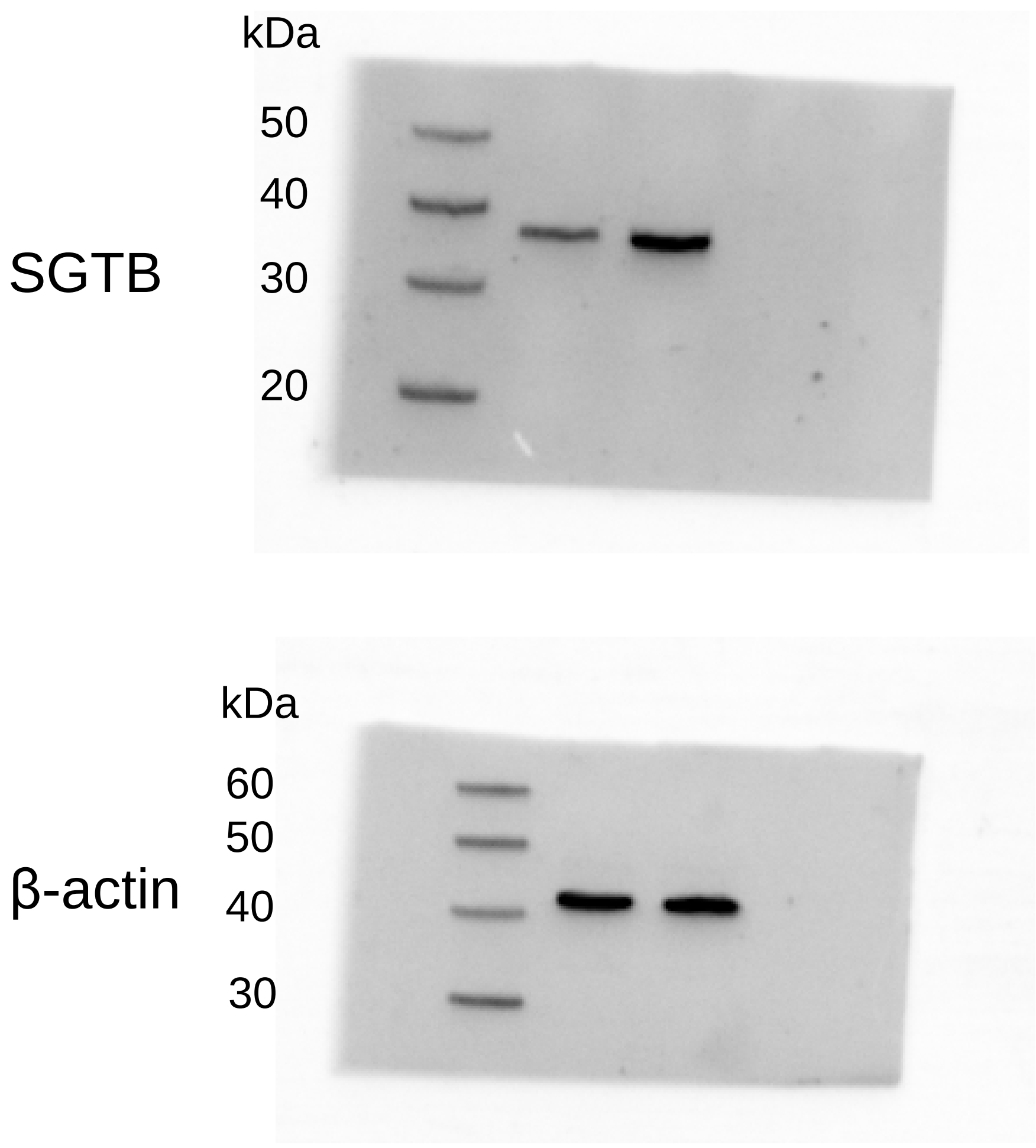

Fig.5I

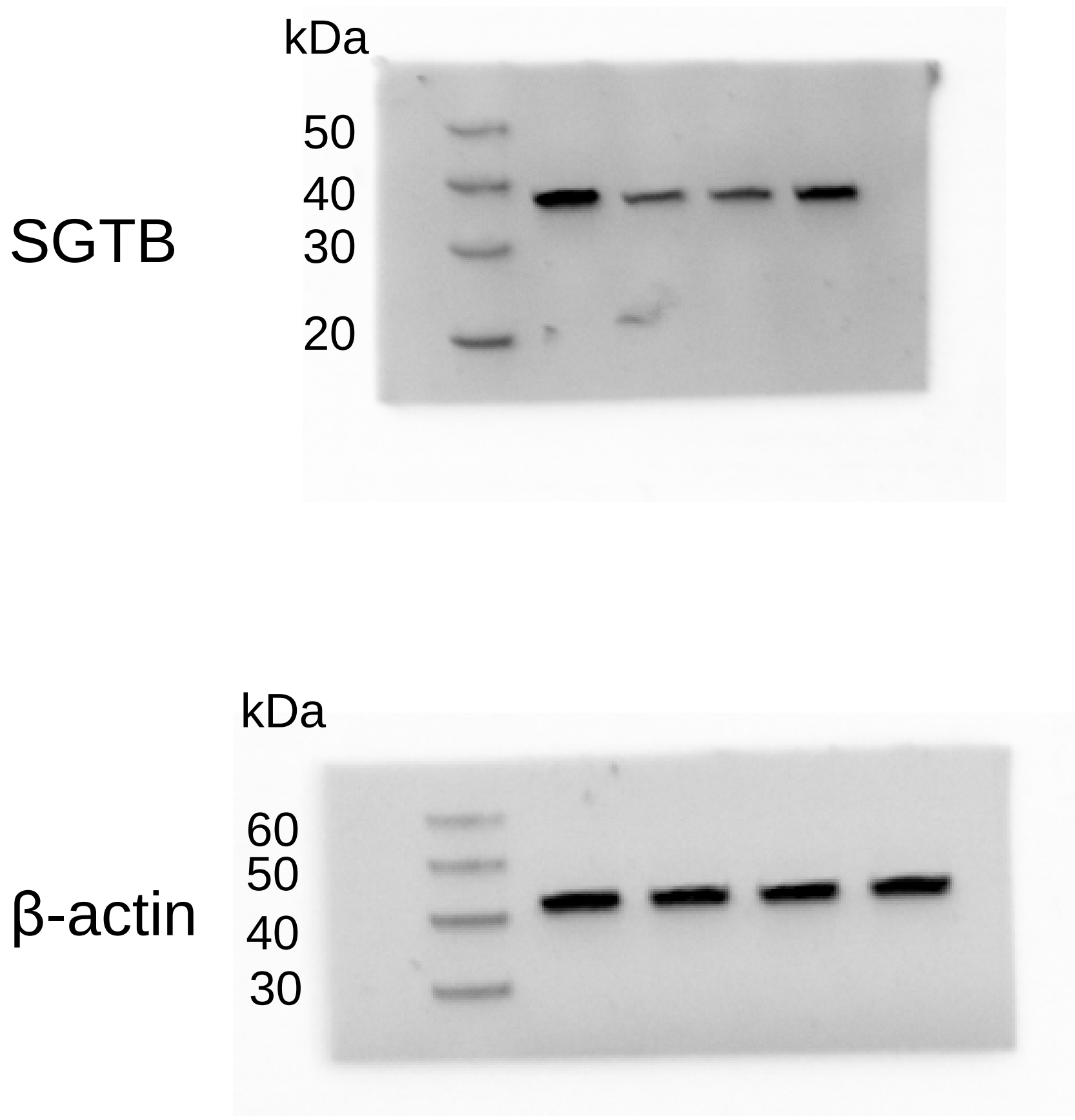

Fig.6C

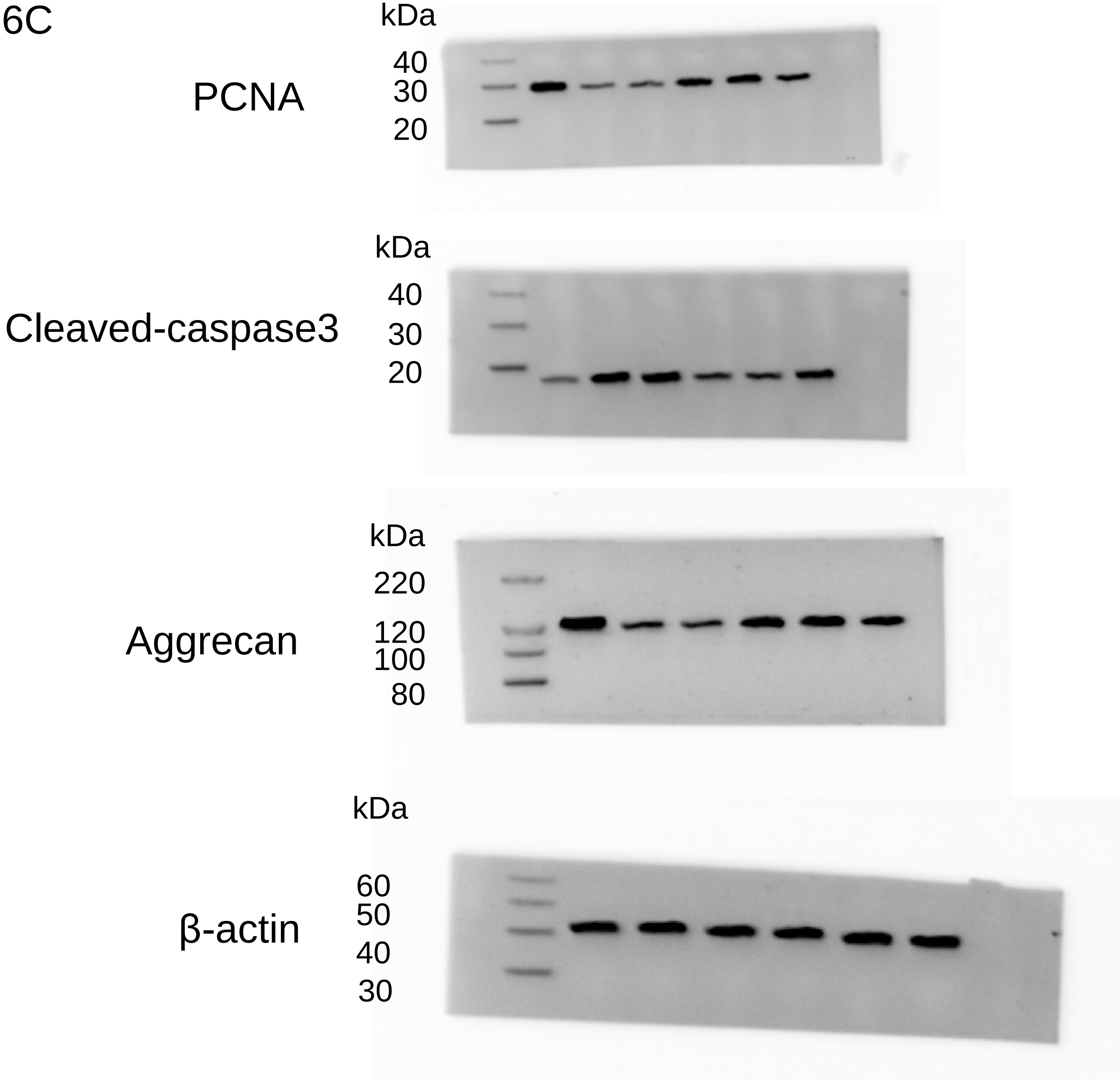
PCNA
kDa
40
30
20
Cleaved-caspase3
kDa
40
30
20
Aggrecan
kDa
220
120
100
80
β-actin
kDa
60
50
40
30

Fig.7B

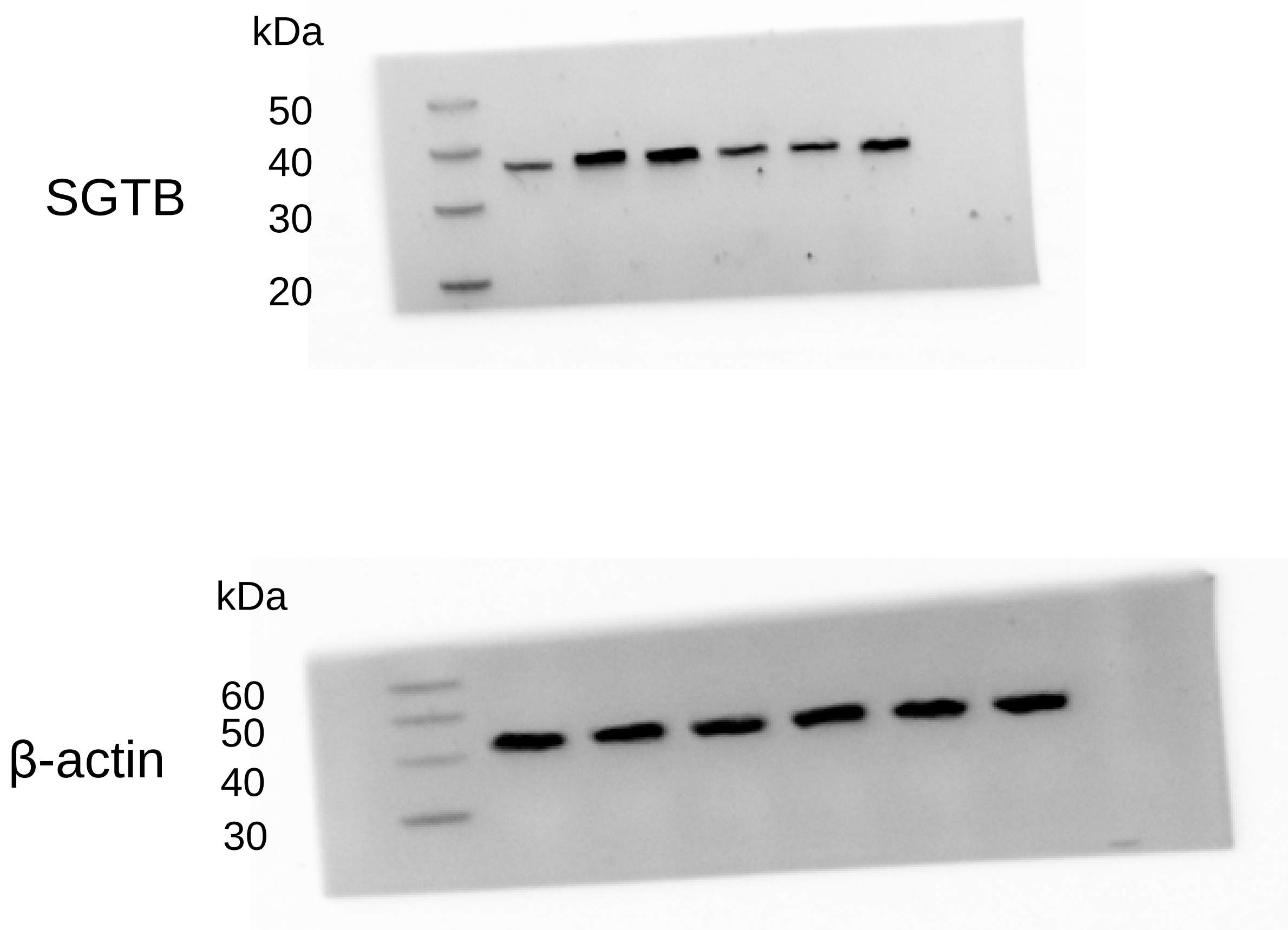

Supplement: Supplementary file 1 [file 42358_2024_429_MOESM2_ESM.pdf]
